# 69567 Association between area deprivation index and long-term diabetic complications in a population of diabetic patients’

**DOI:** 10.1017/cts.2021.716

**Published:** 2021-03-30

**Authors:** Riza C. Li, Kevin Ndura, Claudine T. Jurkovitz

**Affiliations:** Value Institute, ChristianaCare Health Systems

## Abstract

ABSTRACT IMPACT: To improve care and services for patients with chronic disease, health systems are focusing on evaluating social determinants of health of populations at risk; this information is currently not available in electronic health records (EHR) but we show that it could be accessed by linking area deprivation index to EHR. OBJECTIVES/GOALS: To inform care delivery and policy, health care systems are studying ways of improving social determinants of health (SDoH) in patients with chronic disease such as diabetes (DM). Our goal was to better characterize the SDoH of a cohort of DM patients by using the area deprivation index (ADI). METHODS/STUDY POPULATION: Our study population included DM patients seen in primary care practices in 2013-2017. We integrated ADI levels to data extracted from electronic health records (EHR). ADI ranks neighborhoods by socioeconomic status calculated from income, education, employment and housing quality. ADI has 10 levels that we grouped into 5 categories of 2 levels. Addresses were geocoded using ArcMap to obtain census block groups information. We used multivariable logistic regression to calculate odds ratios (OR) and 95% confidence intervals [], with diabetic complications as a binary dependent variable, ADI levels as the exposure, and demographics, smoking status and number of comorbidities as confounders. RESULTS/ANTICIPATED RESULTS: Our study population included 8,558 patients: 56% were female, 61% white, 31% black, 28% were on Medicare, 66% on commercial insurance, median age was 55 years, 57% never smoked, 10% had no comorbidities, 42% had 3 or more comorbidities, and 37% developed diabetic-related complications. After evaluating collinearity and adjusting for confounders, our multivariable analysis showed that worsening ADI was associated with higher likelihood of complications. Compared to ADI level 1&2 (least disadvantaged), the ORs for patients residing in neighborhoods with ADI levels 3&4, 5&6, 7&8, 9&10 (most disadvantaged) were respectively 1.01 [0.88-1.16), 1.20 [1.04-1.39], 1.15 [0.99-1.33], 1.30 [1.11-1.52]. DISCUSSION/SIGNIFICANCE OF FINDINGS: Neighborhood ADI could provide precious information to health care providers when associated to the EHR. We found that neighborhoods with ADI level 9&10, which is not collected in the EHR, was significantly associated with a higher burden of disease. ADI could serve as a proxy for evaluating SDoH.

